# A novel method for predicting the budget impact of innovative medicines: validation study for oncolytics

**DOI:** 10.1007/s10198-020-01176-x

**Published:** 2020-04-04

**Authors:** Joost W. Geenen, Svetlana V. Belitser, Rick A. Vreman, Martijn van Bloois, Olaf H. Klungel, Cornelis Boersma, Anke M. Hövels

**Affiliations:** 1 Division of Pharmacoepidemiology & Clinical Pharmacology, Utrecht Institute for Pharmaceutical Sciences (UIPS), Utrecht University, Universiteitsweg 99, 3584 CG Utrecht, The Netherlands; 2 National Health Care Institute, Eekholt 4, 1112 XH Diemen, The Netherlands; 3 Brabers, Hogeweg 16, 2585 JD Gouda, The Netherlands; 4 Health-Ecore, 1e Hogeweg 196, 3701 HL Zeist, The Netherlands; 5 Division of Global Health, Department of Health Sciences, University Medical Center Groningen, University of Groningen, Antonius Deusinglaan 1, 9713 AV Groningen, The Netherlands

**Keywords:** Budget impact, Oncology, Medicines, Budget impact estimation, Prediction modeling, Validation study, I180, I130, I100

## Abstract

**Background:**

High budget impact (BI) estimates of new drugs have led to decision-making challenges potentially resulting in restrictions in patient access. However, current BI predictions are rather inaccurate and short term. We therefore developed a new approach for BI prediction. Here, we describe the validation of our BI prediction approach using oncology drugs as a case study.

**Methods:**

We used Dutch population-level data to estimate BI where BI is defined as list price multiplied by volume. We included drugs in the antineoplastic agents ATC category which the European Medicines Agency (EMA) considered a New Active Substance and received EMA marketing authorization (MA) between 2000 and 2017. A mixed-effects model was used for prediction and included tumor site, orphan, first in class or conditional approval designation as covariates. Data from 2000 to 2012 were the training set. BI was predicted monthly from 0 to 45 months after MA. Cross-validation was performed using a rolling forecasting origin with e^|Ln(observed BI/predicted BI)| as outcome.

**Results:**

The training set and validation set included 25 and 44 products, respectively. Mean error, composed of all validation outcomes, was 2.94 (median 1.57). Errors are higher with less available data and at more future predictions. Highest errors occur without any prior data. From 10 months onward, error remains constant.

**Conclusions:**

The validation shows that the method can relatively accurately predict BI. For payers or policymakers, this approach can yield a valuable addition to current BI predictions due to its ease of use, independence of indications and ability to update predictions to the most recent data.

**Electronic supplementary material:**

The online version of this article (10.1007/s10198-020-01176-x) contains supplementary material, which is available to authorized users.

## Introduction

In recent years, the prices of new drugs, for example in oncology, have increased considerably [1]. Combined with the increasing number of annual oncology approvals and expanding indications, drug treatment costs in this field have increased sharply. This has resulted in significant macrolevel budget impact (BI) discussions [1]. High BI estimations and the potential of a negative impact on affordability and patient access have, unsurprisingly, led to decision-making challenges and debate [2–5].

In many jurisdictions, patient access is governed by institutional payers or national reimbursement agencies [6]. When facing budgetary constraints, as is the case in, for example, England, (additional) spending on one drug must be covered by disinvesting in other interventions or services [7, 8]. Budgetary limitations and budgeting policies cause payers or reimbursement agencies to limit access to high-priced pharmaceuticals and/or products with a high BI and therefore burden to healthcare budgets and society [9].

It is a trend that more new drugs gain marketing approval with limited evidence packages [10]. The orphan designation and conditional approval legislation might have been successful in increasing the therapeutic options in some disease areas, but it does have adverse effects on payers: much more uncertainty regarding clinical and cost-effectiveness and BI [11–15]. The combination of high price and high uncertainty in (cost-)effectiveness as well as the potential population size and therefore budget impact poses the greatest risk to payers or budget holders. In this study, we will focus on the budget impact as source of uncertainty in reimbursement decision making. According to a review by van de Vooren et al., many published budget impact analyses (BIAs) still fail to reach an acceptable quality [16]. Many BIAs are short term (1 year), quite subjective or based on expert opinion and determined by estimations of population size and eventual treatment regimen [17, 18]. If the general methodological quality of BI analyses is indeed low, one would expect the predictive accuracy of these analyses to also be low.

Broder et al., who evaluated BI forecasts of US drug launches between September 1, 2010, and September 1, 2015, concluded that the average predicted BI was 5.5 times the observed BI. Cha et al. concluded that 60% of the drug forecasts were off by more than 40% [17, 18]. Keeping et al. recently wrote that BI estimates used by Welsh payers that were specifically produced to inform access decisions were off by more than 40% in 80% of the cases [19]. We believe that these findings illustrate that the methodological quality as well as the predictive accuracy of current BIAs can be considered as low.

Not only are these estimations insufficient in providing adequate clarity on the costs of a new drug, but they also fail to quantify the uncertainty that is associated with these predictions. In other words, the current point estimates or ranges given are not based on an underlying probability distribution and thus provide insufficient insight into the possible range of financial outcomes. Especially given the concerns regarding accuracy and methodological quality mentioned previously, insights into uncertainty surrounding BI estimates could prove to be a crucial step in increasing the use and validity of BIA. Consequently, noting that conducting (probabilistic) sensitivity analysis is now standard practice is cost-effectiveness analysis (CEA), allowing for proper sensitivity analysis in BIA could increase its validity.

Proper incorporation of (accurate) budget impact predictions in reimbursement decisions is essential for ensuring patient access and affordability [1–4]. Therefore, we developed a new approach for BI predictions using population-based drug volume data and a mixed-effects model aiming to improve prediction of future BI and quantification of uncertainty of the predicted BI. In this paper, we describe this BI prediction approach and the validation of this method using a Dutch perspective and using oncology drugs as a case study.

## Methods

We used population-level data provided by FarmInform to estimate and validate BI [20]. These data contain the monthly BI as list price multiplied by volume (generated in the in- and outpatient setting) of all prescription drugs in the Netherlands from January 1, 2000, to October 1, 2017. We denote these monthly products as BI data records. FarmInform cross-checks the data with Dutch patient-level PHARMO data to ensure generalizability [21, 22].

Products were selected using the following criteria: First, products should belong to the ‘antineoplastic agents’ (L01) ATC category. Second, products should have gained marketing authorization (MA) by the European Medicines Agency (EMA) between January 1, 2000, and October 1, 2017. Third, the European Public Assessment Report (EPAR) should state that the product received MA for an oncology indication [23]. Finally, the product should be designated as a ‘New Active Substance’ by the EMA [23]. Biosimilars were then excluded.

For all included products, the following characteristics (at time of MA) were collected from the EPAR and/or the European Commission Decision documents [23, 24]: orphan designation, conditional approval, MA under exceptional circumstances, the molecule type (e.g., small molecule, monoclonal antibody) and indication(s). MA under exceptional circumstances and conditional approval were then combined into one covariate denoted as ‘CE.’ Indications were subsequently categorized into cancer sites (e.g., breast, lung). We furthermore collected data on Food and Drug Administration (FDA) First in Class (FiC) designation which we derived from Eder et al. and FDA Novel Drug Approvals summaries [25, 26]. As we chose the perspective of individual drug products and not drug classes or patient populations, indication extensions or label changes of products were not included.

We used a mixed-effects model for prediction. Model building and validation were performed in R for Windows using the nlme package [27, 28]. The dataset was split in a training set and a validation set based on the date of the monthly BI data record. The training set was used for constructing the mixed-effects model. The splitting point of the dataset for model building was set at 149 months, indicating that products with a BI data record prior to May 1, 2012, were selected as training set and products with a first BI data record after this date as validation set. Only the first 45 months of BI records per drug was included as this is the period we aim to predict, denoted as *t_max.* The duration of the period for the training set was based on a proper balance between the number of products in the training set (*n* = 25) and an adequate number of months in the validation set. This adequate number of months of data in the validation set is needed to ensure the capture of a sufficiently large portion of the 45 months of data for a sufficient amount of validation set products (*n* = 44).

On the training set, model building was performed using forward stepwise selection (lowest Akaike information criterion (AIC), *p* < 0.10). Interactions with the square root of time and time to the power 1–6 were included as a possible step to model time dependence. Only a single time interaction per covariate was allowed.

As Shmueli stated, overfitting to training data is the biggest danger to generalizability of predictive models [29]. Moreover, it is explained that it is not required to explore the causal structure of variables as, in prediction models, predictor selection should be solely based on quality of the association between the predictor and response [29]. In order to limit risk of overfitting, we therefore did not force main effects of interactions to be included in the model. Due to right-skewed BI data, log transformed monthly BI (per lowest AIC) was selected as dependent variable. Random effects were composed of a random intercept and a random slope for time per product, based on lowest AIC. The correlation structure was defined as autoregressive with an order of 1 for time.

**Fig. 1:**
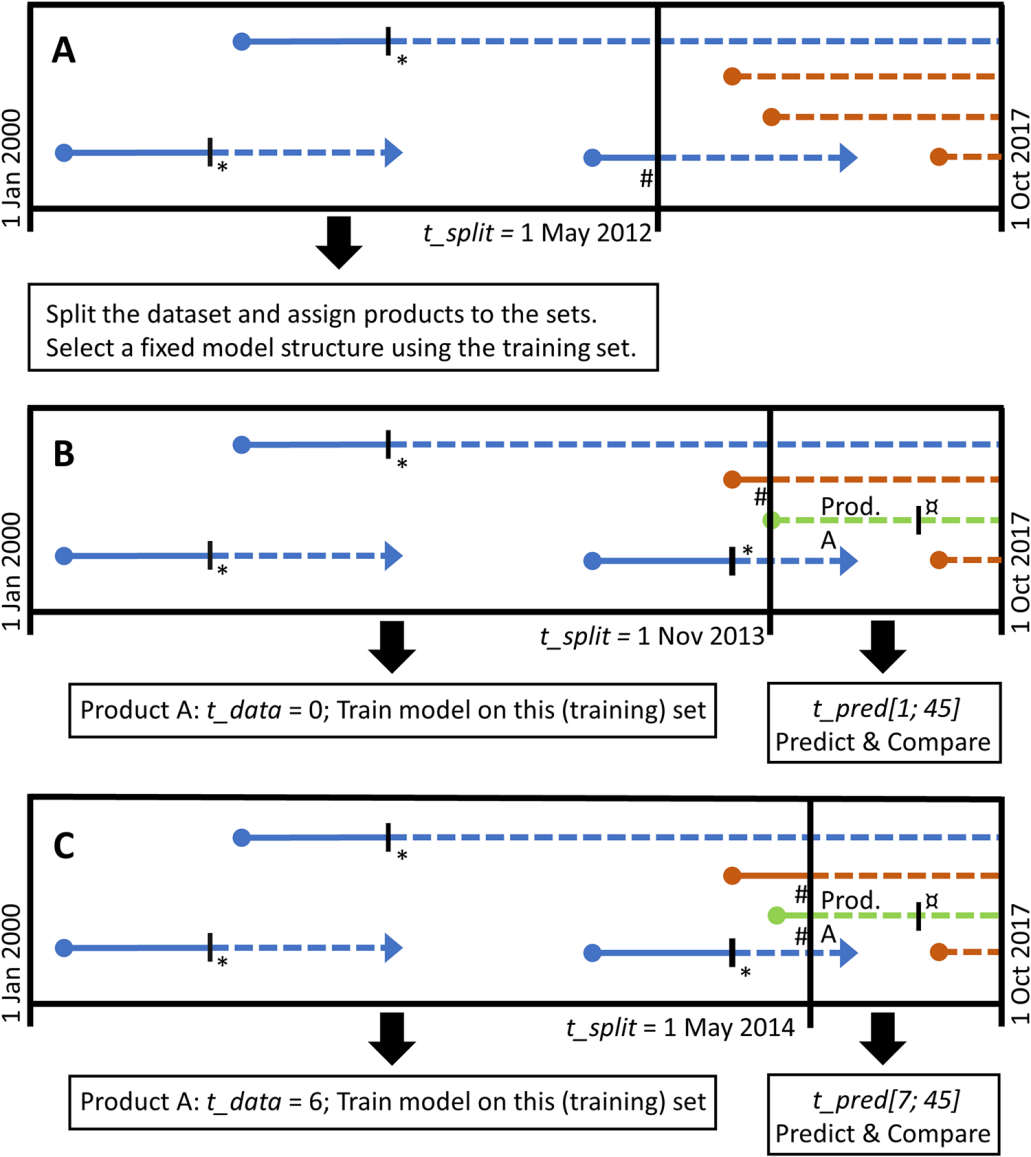
Schematic presentation of the role and construction of the training and validation set, model development and the validation using a rolling forecast origin. Arrows indicate BI record availability of a specific product. A dashed line indicates trimmed data, and a solid line indicates data included in a training set. Blue is assigned to training set products, orange to products that will be validated and green to the product that, in this example, is validated. * denotes data cutoff based on *t_max*. # denotes data cutoff based on *t_split*. ¤ denotes the maximum value of *t_pred* which is identical to *t_max*. **a** Training set selection for model development and resulting selection of validation products, **b** validation of the product depicted in green with *t_data* = 0. The *t_split* = November 1, 2013, similar to the first date of recorded BI for this particular product, **c** validation of the product depicted in green with *t_data* = 6. The *t_split* = May 1, 2014

We then performed cross-validation. Let *A* be the validation set products, *k* be a single product selected from *A* and *B* be the resulting list of products in the training set which does not include *k. A* is constructed by selecting all products with a first BI record after May 1, 2012. Figure 1a–c provides a schematic overview of the validation procedure.

We simulated the effect of the monthly addition of new data, thereby modeling the passing of time and the influence this has on prediction by using a rolling forecasting origin. Hence, we adapt the training set to include all BI records on *B* just 1 month prior to the date of the first BI record of *k*. *t_data* represents the number of months of data available to model building and prediction, while *t_pred* indicates the month which is predicted. *t_split* governed the rolling forecast and indicates the date at which the dataset is split into training and validation set.

The rolling forecasting origin, simulating passing time, used the following procedure: We set the initial cycle to start with zero BI records (*t_data* = 0) on *k; t_split* is set to the date of the first BI record of *k*. The training set is constructed to include all data until *t_max* (45 months) and *t_split* on *B*. The first 45 months (or less if *k* has a shorter MA period) of BI is then predicted for *k* (*t_pred* [1, *t_max*]) and compared with the observed BI data of *k*. In the next cycle, the first month of BI data will become available to the prediction model, so *t_data* = 1 and *t_split* are increased by 1 month. This implies that the first month of *k*’s records is added to the training set with *B,* governed by *t_split*, also advancing 1 month. Prediction and comparison with observed data are then performed for *k* for *t_pred* [2, *t_max*]. The sequence is repeated for *t_data* [2, *t_max* − 1] and all products in *A*. This yields a total of 45 + 44 + … = 1035 time points.

To improve robustness, a validation is also performed on a training set with *t_max* = 42 which includes separate model building on this second training set. Subsequently, validation is performed with 42 months of BI prediction. The mean of the absolute individual predictions is then calculated for all *k* with *t_data* [0, *t_max* − 1] and *t_pred* [1, *t_max*] by taking the average of these data points for the 42 and 45 *t_max* runs. This produces the final prediction results for each *k* with a specific *t_data* and *t_pred,* denoted as the prediction samples.

The previous paragraphs have outlined the role of the training set (selection of model structure) and the validation set (accuracy of predictions, given the chosen model structure). Coefficients are, unlike the traditional notion of a training set, not governed by the initial training set, but are estimated for each prediction cycle, based on the available data (governed by *t_split*). We thus aim to validate a BI prediction approach which uses a fixed model structure and where the model is continuously retrained on future data. This validation approach is adopted as it represents the envisioned implementation that can adapt to patient and market dynamics.

We capped predicted BI to a minimum or maximum value in order to limit the effect of potential outliers. The maximum predicted monthly BI was determined as two times the maximum monthly BI in the total dataset. The minimum monthly BI was set at an arbitrary €5000. The influence of limiting these values is explored by means of scenario analysis.

Equation 1 describes the calculation of the prediction error.

1 $$ {\text{error}} = e^{{\left| {\ln \frac{{{\text{Observed}}\;{\text{BI}}}}{{{\text{Predicted }}\;{\text{BI}}}}} \right|}} $$

The resulting ratio is symmetric for over- and underprediction [30, 31]. The purpose of this transformation is to yield ratios that have a positive sign for over- as well as underprediction so that overpredictions do not cancel out underpredictions. An error of 2 should therefore be interpreted as, in case of observed BI of €10,000, a predicted BI of €5000 or €20,000. This error was calculated for each prediction sample, yielding error samples.

Results were compiled in three ways:

1. Aggregated per *t_pred* and *t_data*: Error samples are aggregated for each point in (*t_pred*, *t_data*).
2. Aggregated per *t_data*: Error samples are aggregated for each *t_data*.
3. Not aggregated: Outcomes on all individual error samples.

In order to compare our results to the previously published literature, we calculated the percentage of predictions that are between 40 and − 40% and between 100 and − 100% of the observed BI. Per prediction, this percentage is calculated using Eq. 2:

2 $$ {\text{percentage}}\;{\text{difference}} = \frac{{{\text{predicted }}\;{\text{BI}} - {\text{observed }}\;{\text{BI}} }}{{{\text{observed }}\;{\text{BI}}}}*100 $$

For all products, we investigated whether a reimbursement dossier was published by the Dutch Healthcare Institute (ZIN), the authority that performs Health Technology Assessment (HTA) and advises the Dutch Minister of Health on reimbursement of new drugs. For the products with a reimbursement dossier, the amount of *t_data* on the date of publication of the report was recorded. Products can have *t_data* prior to the publication of the dossier when it has been available for another indication or through an alternative access scheme. In our envisioned implementation, the available *t_data* just prior to publication of the dossier would be used to make an up-to-date BI prediction for the product in question.

## Results

The training set used for model building contained 25 products with a mean of 33 months of data, and 15 and 16 products had data until *t_max* of 45 and 42 months, respectively. The validation set included 44 products with an average of 27 months of data and 11 and 14 products having data until *t_max* of 45 and 42 months, respectively. This resulted in a total of 19,681 prediction and error samples. The products included in the datasets are displayed in Supplemental Table 1.

Fixed-effect selection for the 42 and 45 *t_max* models yielded the same fixed effects being time + time * CE, √(time) * Tumor site, molecule type, √(time) * FiC and √(time) * orphan designation. As random effects were not varied, both model structures are identical. The final model syntax was:

$$ \begin{aligned} & [{\text{lme}}({\text{fixed}} = { \log }({\text{observed}}\;{\text{BI}})\sim{\text{Time}} + {\text{Time}}:{\text{CE}} + {\text{Molecule}}\_{\text{type}} \\ & \quad \quad + {\text{sqrt}}\left( {\text{Time}} \right):\left( {{\text{Orphan}}\_{\text{status}} + {\text{FiC}}\_{\text{status}} + {\text{Tumor}}\;{\text{site}}} \right),\;{\text{random}} \\ & \quad = \sim{\text{Time}}|{\text{Product}},\;{\text{correlation}} = {\text{corARMA}}\left( {p = 1,\;q = 0,\;{\text{form}} = \sim{\text{Time}}|{\text{Product}}} \right)] \\ \end{aligned} $$

**Fig. 2:**
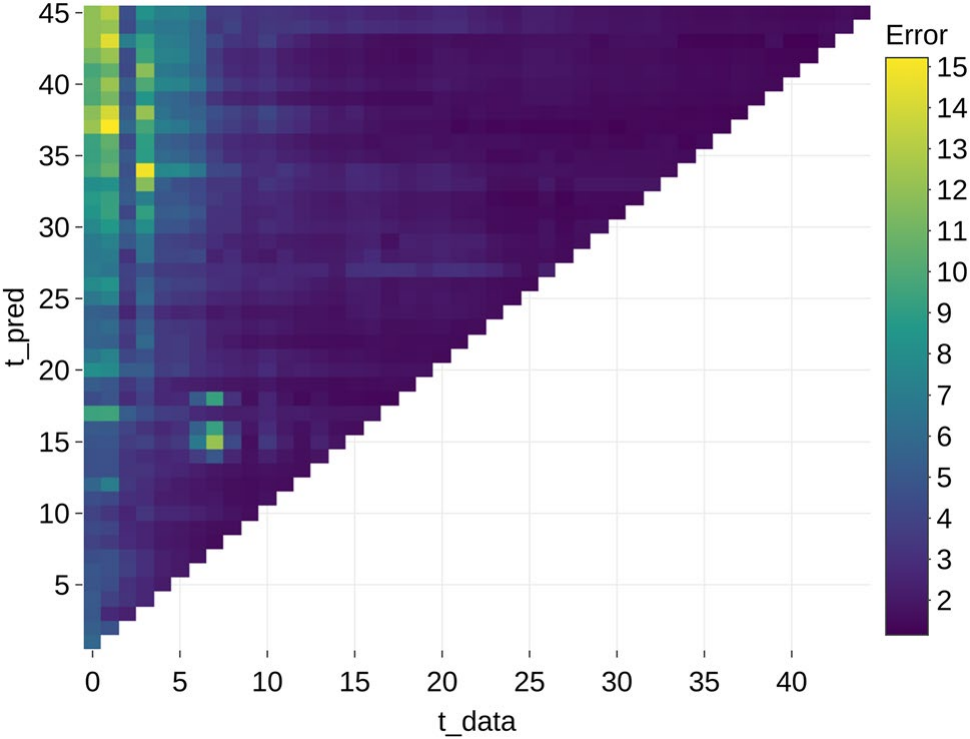
Mean error aggregated per future month (*t_pred*) and available data (*t_data*)

The results that are aggregated per *t_pred* and *t_data* are shown in Figs. 2 (mean) and 3 (median). These figures illustrate that the errors are higher in models with less available data (low *t_data*) and at predictions further in the future (a higher *t_pred*). The highest errors occur in the models without any prior data (*t_data *= 0) with a mean error ratio of 6.37. From *t_data *> 10, the error seems to remain constant.

**Fig. 3:**
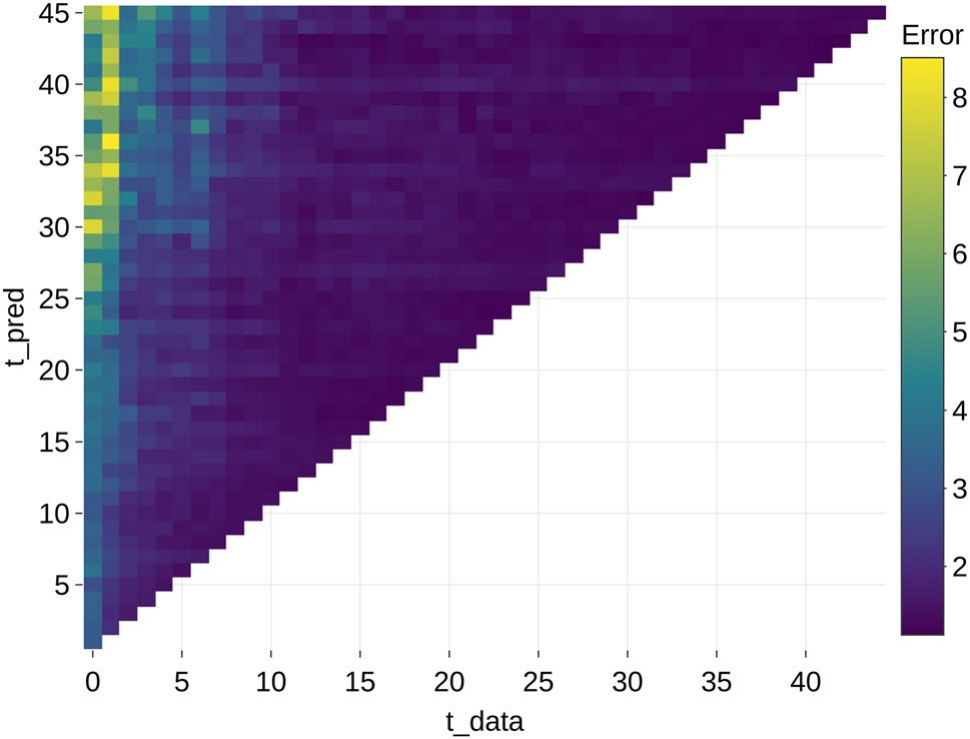
Median error aggregated per future month (*t_pred*) and available data (*t_data*)

**Fig. 4:**
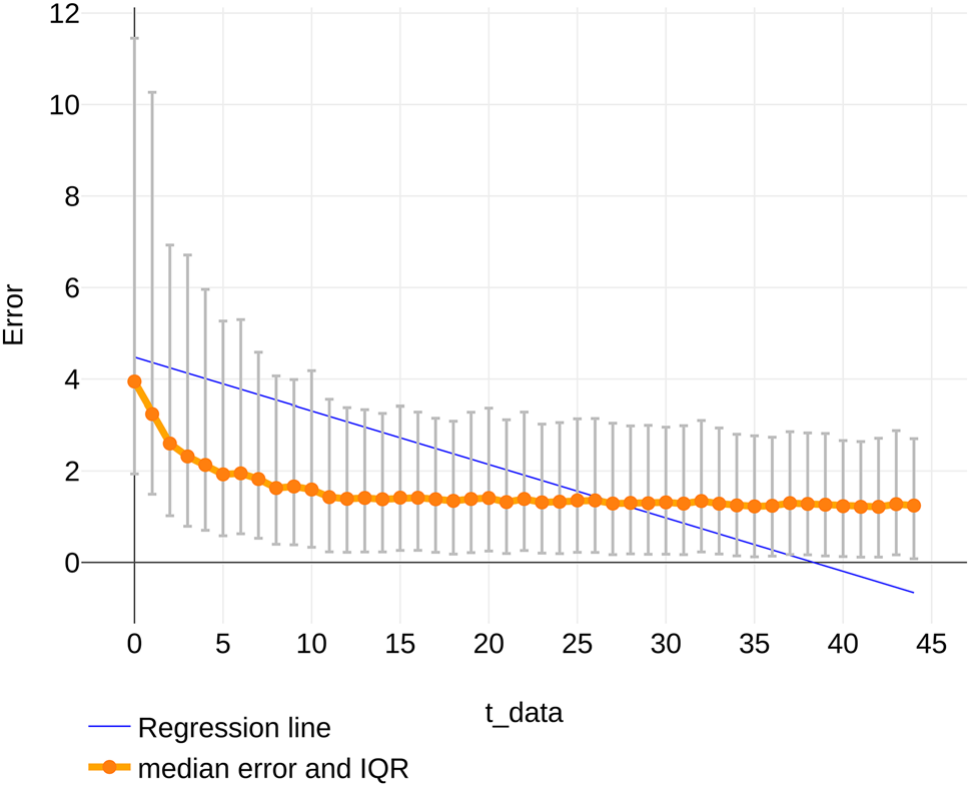
Median error (orange) aggregated per *t_pred*, including error bars indicating the interquartile range and the regression line (blue). Coefficient = − 0.096, se = 0.0035, *p* < 0.0001

In Fig. 4, we present the results that are aggregated per *t_data*. The errors are clearly left-skewed and significantly reduce with increasing *t_data* as established using a linear regression on the individual samples (coefficient = − 0.117, se = 0.0039, *p* < 0.0001). The interquartile range (IQR) decreases with increasing *t*_*data*. Prediction performance increases substantially from increasing *t_data* from 0 to 5; from *t_data* > 10, model accuracy does not improve by adding more data.

**Fig. 5:**
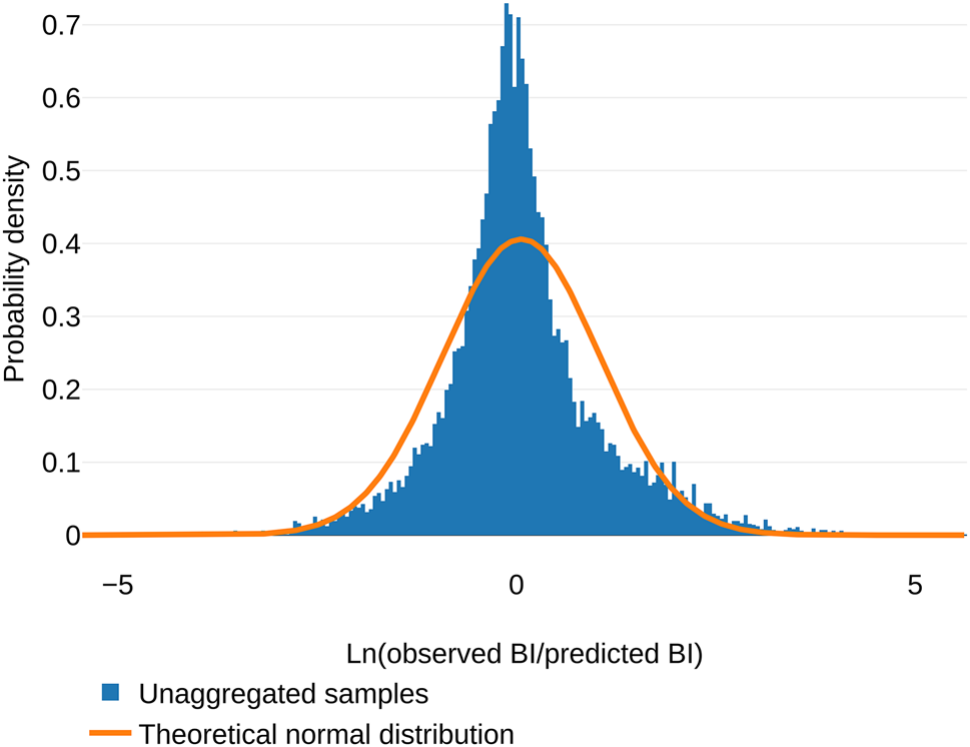
Histogram of the individual outcomes. Outcomes calculated as Ln(observed BI/predicted BI) (blue) and the theoretical normal distribution (orange)

The unaggregated mean and median error for all samples are 2.94 (standard deviation (SD) = 5.64) and 1.57 (interquartile range (IQR) = 1.42), respectively. The mean of Ln(observed BI/predicted BI), so without converting to absolute values, of all samples, depicted in Fig. 5, did significantly differ from 0 (*t* test: mean = 0.057, *n* = 19,681, 95% CI = 0.043;0.070, *p* < 0.0001). In absolute terms, underprediction was significantly more likely than overprediction (exact binomial test, prob. underprediction = 0.515, 95% CI = 0.508; 0.522, *p* < 0.0001). Using Eq. 2, we calculated the percentage difference that can be compared with other literature. Of the 19,681 samples, 9,503 (48.3%) had a maximum percentage difference between 40 and − 40%. For the 100% to − 100% range, this number was 15,915 (80.9%). Table 1 summarizes the main outcomes.

**Table 1:** Main validation outcomes

Outcome	Value
Mean error, aggregated per *t_data* and *t_pred* (SD)	3.01 (2.24)
Mean error, not aggregated (SD)	2.94 (5.63)
Median error, not aggregated (5th, 25th, 75th and 95th‰)	1.57 (1.04, 1.21, 2.63, 8.59)

In the training set, three products did not have a reimbursement dossier and nine products did not have *t_data* at the time of publication of the dossier. For the 13 training set products with *t_data*, the median and mean months of *t_data* were 22 and 29.8 (sd = 32.1), respectively. In the validation set, 28 products did not have a dossier and 10 products (with a dossier) had 0 *t_data*. The six products with a dossier and *t_data* > 0 had a median and mean *t_data* of 12.5 and 16.5 (sd = 11.8), respectively.

### Scenario analyses

We explored the influence of limiting predictions to a minimum (< €5000) and maximum (> 2 times maximum recorded BI) on the outcomes by adopting scenarios where (1) only minimum values were adjusted, (2) only maximum values were adjusted and (3) no predictions were adjusted. In the base case analysis, on a total of 19,681 samples, 930 minimum and 44 maximum values were adjusted. The outcomes are presented in Supplementary Tables 2–4.

These results indicate that not limiting minimum values has a profound negative influence on model performance as, presented in Supplementary Table 3, but mean unaggregated error increases to 8.00 with a very large sd of 358.74. On the contrary, not limiting maximum only has a minor impact, as Table 1 and Supplementary Table 2 yield nearly identical results. As only the more extreme values are concerned, it is logical that the median figures (Supplemental Tables 2–4) are very similar to the median base case results.

## Discussion

Our prediction model was constructed using a mixed-effects approach with a training set of 25 oncology products. The validation of this prediction model using a training set of 44 products yielded 19,681 samples and resulted in an overall mean error of 2.94. This error is higher when available data are more limited and when predicting further into the future. The decline in error with increasing available data seems to halt, at a median error of ± 1.5, around 5–10 months of data. This indicates that relatively accurate predictions can be generated with 5–10 months of data. There is a slight but significant higher probability of underprediction versus overprediction. The percentage of predictions that were within 40% to − 40% and within 100% to − 100% of observed BI were 48.3% and 80.9%, respectively.

We envision the following implementation: Initially, a model structure would be selected and validated using the procedures herein described. Then, with an estimate of the model performance, BI predictions, using up-to-date data of all other products based on which the model is trained, can then easily be generated for a new product (with or without prior BI data of that product). The validation results can yield insights into the expected accuracy for this new drug for a specific future month (*t_pred*) and a specific amount of available BI data (*t_data*). At some future moment, of which the specifics are beyond the scope of this paper, continuous model retraining on new data will probably not suffice as the validation set at that time will not be representative of the training set on which the model structure was developed. In that case, model selection and validation would have to be redone. This would also be applicable to using our methodology in other jurisdictions or geographic areas, for predicting different or entirely new drug classes or when adapting to changes in regulatory systems.

Our BI predictions are quite constant and rather accurate from 10 months of available data as the median aggregated error from 10 months onward ranges from 1.20 to 1.42. As the error is highest with little available data, one could say that our approach is not useful for BI predictions when these predictions are part of a reimbursement dossier in a ‘closed’ reimbursement system, indicating reimbursement for new drugs is only available after an HTA decision. However, various countries have (partly) open systems (e.g., Germany, the Netherlands) wherein HTA dossiers become available after the drug is already available and in use. In our dataset, we have shown that 50% of the products have a substantial amount of data available at the date of publication of the reimbursement dossier. It is therefore very probable that, at least for open reimbursement systems, a sufficient amount of BI data will in many cases be available to overcome the high errors associated with having less than 5–10 months of available data for prediction.

When extending the use of BIA beyond the initial reimbursement decision to a more dynamic drug life cycle approach, for example as part of managed entry agreements, available BI data will keep increasing and will therefore rapidly be sufficient for achieving our reported maximum predictive accuracy [32].

Cha et al. analyzed the accuracy of peak sales forecasts produced by the so-called sell-side analysts [17]. They categorized the forecasts in categories of percentage difference between forecasts and observed peak sales. Their highest deviation categories were < − 80% (*n* = 7/260) and > 160% (*n* = 57/260) and found a median error of 4%. They do state that most forecasts are poor and that the variance is high, but the 4% median error does not give clear insight into forecast error as overestimations can cancel out underestimations (i.e., their error is not symmetric) and as the maximum error is limited (− 80% and 160%). We have partly applied the methodology used by Cha et al. to our dataset by also limiting the maximum error and by not making the error symmetric. Using this method, our median error is − 3%, a major difference between our unaggregated and symmetric median error of 1.54 (154%). Cha and colleagues furthermore state that more than 60% of the forecasts were off by more than 40%, whereas in our analysis 51.7% of estimations had a higher deviation than 40%.

Broder et al. published a review of the bias in BI predictions of new drugs [18]. They used a US perspective and included formal, more scientific, BI predictions as well as informal predictions that are aimed at projecting share prices. All estimates were made less than 12 months before launch, and nearly all estimates were for just the first year. Mean predicted BI in the sample was 5.5 times the observed BI. When excluding the informal predictions, the average overestimation is 5.6 times the observed BI. These values are asymmetric representations of under- and overprediction (i.e., the value is attenuated toward 1 due to underpredictions that have a value between 0 and 1) and are still higher than our (symmetric) mean error. Only 20% of the predictions were within 40% of the observed usage, compared to 48,3% for our model [19]. If we then relate to the differing lengths of forecast period (i.e., *t*_*pred*) of 1 year for Broder et al. and 45 months for our study, we could argue that our predictions seem to have better accuracy while providing more future predictions.

Keeping et al. investigated BI estimates that were part of pharmaceutical company submissions and compared them to the observed expenditure [19]. These company submissions were issued to the All Wales Medicines Strategy Group (AWMSG) for reimbursement decision making. The AWMSG is the institution that appraises clinical and cost-effectiveness of new medicines being considered for the National Health Service prescribing in Wales (UK). A total of 49 medicines were included, and the percentage of predictions in the 40% and 100% range was 20.4% and 53.1%, respectively. Our model achieved 48.3% and 80.9% on these respective accuracy markers. Of the 49 products Keeping and colleagues included, only 3–6 (depending on the definition) had an oncological indication which is therefore quite different from our oncology cohort. Still, as the BI estimates investigated by Keeping et al. are those used by payers to inform decision making, the work of Keeping et al. is very relevant. Even though our results are not directly comparable, we still argue that our superior performance in the 40% and 100% range metric is a rather clear indicator that our method has the potential to be superior to current BI estimates used by payers and decision makers.

Our BI prediction approach potentially has several advantages over current BI estimation procedures. Firstly, our methodology is independent of indication extensions. Of course, additional indications do have an influence on BI and possibly on the accuracy of the predictions. We, however, chose to not include indication expansions as a predictor variable as this would be rather laborious to perform in practice for a large group of products. Unlike current Dutch Reimbursement authority (‘National Health Care Institute’) BI predictions, our model intrinsically adjusts for possible changes in indications as we apply a drug perspective irrespective of indication.

Another potential advantage of our BI prediction approach is the ease and speed with which BI predictions can be constructed. Updating the data, possibly performing a separate validation and then performing the prediction for a new drug would be a matter of hours, whereas the current guidelines call for a much more time consuming endeavor [33, 34]. This advantage is especially profound if you include the option of semiautomatically updating the predictions as time passes and more data become available.

Finally, our model results yields predictions with a potentially quantifiable amount of uncertainty as the distribution of error is known and can be adjusted for the amount of data already available (*t_data*) and the number of future months (*t_pred*). This is hardly possible with current BI predictions that produce point estimates. Our approach could therefore serve as a basis for more profound modeling of uncertainty around BI predictions.

Our study has various limitations. First, we have only validated our model for a rather specific set of products and characteristics. Future products, for example novel advanced therapy medicinal products, are not validated and are therefore probably not accurately predictable by our current model. As is, however, described above, the dataset can be updated to future states and a new validation can then be done rather easily in order to accommodate new drug classes and/or characteristics.

Second, we have no direct comparison of our results to the current BI estimations used in practice. As our model is based on Dutch data, it would be very insightful to compare our results with observed BI predictions published by the Dutch Reimbursement authority. In light of the Broder et al., Cha et al. and Keeping et al. findings, our prediction accuracy appears to be superior.

Third, we have capped minimum and maximum BI predictions which to an extent impacted results. Our explicit assumption of a predicted maximum of two times the maximum monthly BI in the total dataset has no evidentiary basis. Potentially worse, there were records in the dataset with monthly BI below the minimum monthly amount of €5000. In other words, it is quite probable that we overestimate certain products with monthly BI below €5000. We, however, believe that these caps are justified as one of our main aims is to provide payers with better BI predictions. A difference between €5000 and €50 yields 4.6 log units of deviation, but this difference, in absolute terms, is probably not very relevant to payers.

We have explored the influence of these value restrictions through scenario analyses. These have clearly indicated that only limiting the lower values to €5000 and not restricting maximum values delivers a nearly identical predictive performance. We thus believe that these limits improve the relevance of our outcomes as prediction errors that are irrelevant on a macrolevel, e.g., €5 versus €5000 per month, are omitted. The high-level caps are implemented as some modeling scenarios yielded predictions that were irrationally high (for example, higher than the entire Dutch healthcare budget) and can therefore be identified by potential users of this method. In order to limit these scenarios to realistic figures, the factor two limit was imposed.

Fourth, we understand that alternative potentially more advanced validation techniques have been developed. In order to construct a methodology that is suitable for informing decision making, the method has to be interpretable and transparent. We therefore abstain from adding more complexity to the current model in order to also keep it as practical as possible.

Still, we believe that, based on our validation, we have developed a valid method to predict BI. We were able to compare our results with three independent studies using a metric that describes the number of predictions that are within 40% to − 40% and 100% to − 100% of the observed BI. Our model was superior to all these three studies, and in particular, the study of Keeping et al. is important in this regard as they investigated the accuracy of BI predictions used by payers for reimbursement decision making.

## Conclusions

The herein presented BI prediction approach can be used to develop models that can provide improved predictive accuracy compared to the current practice of conducting BIA. Additionally, our data-driven approach would allow for a more dynamic, life cycle approach to predicting and managing BI of drugs. To conclude, we think that our approach can be a valuable addition to BI predictions due to its potential for increased accuracy, independence of indications and ability to keep updating the predictions to the most recent data.

## Electronic supplementary material

Below is the link to the electronic supplementary material.

Supplementary material 1 (DOCX 14 kb)

Supplementary material 2 (DOCX 12 kb)

Supplementary material 3 (DOCX 12 kb)

Supplementary material 4 (DOCX 12 kb)
