# Supplementary material for: A novel method for predicting the budget impact of innovative medicines: validation study for oncolytics
Source: Eur J Health Econ. 2020 Apr 4;21(6):845–53. doi: 10.1007/s10198-020-01176-x (PMC7366590; doi:10.1007/s10198-020-01176-x)
Supplement: Supplementary file 1 — Supplementary material 1 (DOCX 14 kb) [file 10198_2020_1176_MOESM1_ESM.docx]

Supplemental Table 1: list of included products in the training set and validation set.

| **Training set product name** | **Training set active substance name** | **Validation set product name** | **Validation set active substance name** |
| --- | --- | --- | --- |
| Xeloda | capecitabine | Adcetris | Brentuximab |
| Avastin | bevacizumab | Zaltrap | aflibercept |
| Vectibix | panitumumab | Tagrisso | osimertinib |
| Erbitux | cetuximab | Inlyta | axitinib |
| Tyverb | lapatinib | Perjeta | pertuzumab |
| Glivec | imatinib | Venclyxto | venetoclax |
| Halaven | eribulin | Zykadia | ceritinib |
| Iressa | gefitinib | Erivedge | vismodegib |
| Votrient | pazopanib | Ibrance | palbociclib |
| MabCampath | alemtuzumab | Lartruvo | olaratumab |
| Nexavar | sorafenib | Pixuvri | pixantrone |
| Jevtana | cabazitaxel | Imbruvica | ibrutinib |
| Arzerra | ofatumumab | Teysuno | tegafur / gimeracil / oteracil |
| Tarceva | erlotinib | Jakavi | ruxolitinib |
| Sutent | sunitinib | Portrazza | necitumumab |
| Tasigna | nilotinib | Kadcyla | trastuzumab |
| Torisel | temsirolimus | Alecensa | alectinib |
| Alimta | pemetrexed | Lynparza | olaparib |
| Sprycel | dasatinib | Imlygic | talimogene laherparepvec |
| Velcade | bortezomib | Caprelsa | vandetanib |
| Vidaza | azacitidine | Mekinist | trametinib |
| Yervoy | ipilimumab | Keytruda | pembrolizumab |
| Zelboraf | vemurafenib | Yondelis | trabectedin |
| Herceptin | trastuzumab | Zydelig | idelalisib |
| Targretin | bexarotene | Opdivo | nivolumab |
|  |  | Kyprolis | carfilzomib |
|  |  | Cyramza | ramucirumab |
|  |  | Darzalex | daratumumab |
|  |  | Dacogen | decitabine |
|  |  | Cotellic | cobimetinib |
|  |  | Vargatef | nintedanib |
|  |  | Ninlaro | ixazomib citrate |
|  |  | Xalkori | crizotinib |
|  |  | Atriance | nelarabine |
|  |  | Gazyvaro | obinutuzumab |
|  |  | Blincyto | blinatumomab |
|  |  | Lonsurf | trifluridine / tipiracil |
|  |  | Giotrif | afatinib |
|  |  | Stivarga | regorafenib |
|  |  | Tafinlar | dabrafenib |
|  |  | Bosulif | bosutinib |
|  |  | Lenvima | lenvatinib |
|  |  | Farydak | panobinostat |
|  |  | Evoltra | clofarabine |
