# Supplementary material for: A novel method for predicting the budget impact of innovative medicines: validation study for oncolytics
Source: Eur J Health Econ. 2020 Apr 4;21(6):845–53. doi: 10.1007/s10198-020-01176-x (PMC7366590; doi:10.1007/s10198-020-01176-x)
Supplement: Supplementary file 3 — Supplementary material 3 (DOCX 12 kb) [file 10198_2020_1176_MOESM3_ESM.docx]

Supplemental Table 3: Results of scenario analysis where only maximum outliers were capped.

| **Outcome** | **Value** |
| --- | --- |
| Mean error, aggregated per t_data and t_pred (SD) | 6.02 (42.79) |
| Mean error, not aggregated (SD) | 8.00 (358.74) |
| Median error, not aggregated (5^th^, 25^th^,75^th^ and 95^th^ percentile) | 1.58 (1.04, 1.22, 2.67, 9.09) |
